# Impact of Prenatal Group Healthy Relationship Education on Postpartum Contraception

**DOI:** 10.1089/whr.2022.0104

**Published:** 2023-03-27

**Authors:** Sara E. Mazzoni, Maggie O'Reilly Treter, Jennifer Hyer, Rachel Peña, Galena K. Rhoades

**Affiliations:** ^1^ Department of Obstetrics and Gynecology, University of Washington, Seattle, Washington, USA.; ^2^ Department of Psychology, University of Denver, Denver, Colorado, USA.; ^3^ Department of Obstetrics and Gynecology, Denver Health and Hospital, Denver, Colorado, USA.

**Keywords:** postpartum contraception, healthy relationship education, long-acting reversible contraception

## Abstract

**Objective:**

We aimed to evaluate the impact of an antenatal group healthy relationship education program on the postpartum use of long-acting reversible contraception (LARC).

**Materials and Methods:**

This is a planned subgroup analysis of a larger randomized controlled trial. Pregnant and newly parenting women were randomized to either group healthy relationship education, “MotherWise,” or no additional services. An evidence-based healthy relationship education program and individual case management sessions were provided. The program did not include any prenatal care or contraception counseling. This subgroup analysis included those participants with a nonanomalous gestation randomized at <40 weeks who received care and delivered at a single safety-net hospital and were discharged home with a live infant(s).

**Results:**

From September 2, 2016 to December 21, 2018, 953 women were randomized in the larger trial; 507 met inclusion criteria for this study; 278 randomized to program and 229 controls. Participants were mostly young, parous, Hispanic, publicly insured women. Participants randomized to program were more likely to take a prescription medicine and be delivered through cesarean; there were not any other significant differences in baseline, antenatal, or perinatal outcomes. Those randomized to program were more likely to be discharged home with immediate postpartum LARC in place (odds ratio [OR] 1.87; confidence interval [CI] 1.17–3.00), and more likely to be using LARC at the postpartum visit (OR 2.19; CI 1.34–3.56).

**Conclusion:**

Antenatal group healthy relationship education provided separately from prenatal care is associated with a twofold increase in the use of postpartum LARC.

**Clinical Trial Registration:**

ClinicalTrials.gov NCT02792309; https://clinicaltrials.gov/ct2/show/NCT02792309?term=NCT02792309&draw=2&rank=1

## Introduction

Pregnancy may be considered a window of opportunity when women are motivated to change their health behaviors. Group prenatal care (GPC) is a model of prenatal care developed with the core principles of education, social support, and empowerment—a model, which lends itself nicely to this theory that antenatal interventions can impact not only immediate birth outcomes, but longer term health outcomes for the mother if health behaviors are changed. It is not surprising then that GPC has been shown to improve some postpartum outcomes, most notably the increased use of family planning services postpartum.^1^

Based on similar tenets as GPC, healthy relationship education programs are built on the foundation of teaching skills and tools to improve and maintain healthy intimate partner relationships. Among other things, they have been associated with higher relationship happiness, less physical assault, less psychological abuse, and lower psychological distress.^2^ These programs were traditionally developed and delivered to couples. However, they have also been shown efficacious when offered only to individuals and not both partners.^3,4^ Within My Reach,^5^ one such example, is designed to equip individuals with the skills, tools, and resources they need to make the best decisions for themselves and their families and has been shown to improve relationship skills and other family outcomes.^6–8^

It stands to reason then that an intervention during pregnancy aimed at empowering and educating women about healthy relationships would increase positive health behaviors postpartum—even when those health behaviors are not directly addressed by the intervention. Based on this theory, we developed a novel program combining the principles of GPC and healthy relationship education, “MotherWise.” The MotherWise program provides group healthy relationship education (Within My Reach) and one-on-one case management and does not provide any prenatal care. Our primary objective of this subanalysis was to evaluate the impact of MotherWise—when offered separate from routine prenatal care—at any time during pregnancy on postpartum use of effective contraception.

## Materials and Methods

This study is a planned subgroup analysis of a larger randomized controlled trial in which participants were randomized to either a group healthy relationship education program, “MotherWise,” or no additional services from September 2, 2016 to December 21, 2018. Pregnant and newly parenting women were recruited from prenatal care visits at a safety-net hospital, as well as from the community through social media, radio, and social service referrals. Randomization was initially 3:2 to create groups of adequate numbers in the intervention arm, then changed to 1:1 when recruitment was sufficient after 7 months.

An evidence-based healthy relationship education curriculum, Within My Reach, as well as brief information on infant care and parenting was provided across six weekly 4-hour group classes (24 hours total of curriculum). The curriculum included information on what healthy relationships are like, ways to leave unsafe relationships, skills for good communication and conflict management, as well as information we developed for MotherWise specifically on connecting with and caring for a newborn. A central theme of Within My Reach is “sliding vs. deciding,” a concept^9^ that encourages making empowered decisions rather than sliding into circumstances that may make a person feel stuck or lead to negative consequences, such as an unintended pregnancy. Groups were offered in English and Spanish and typically had 6 to 12 women. Within My Reach is a manualized intervention. Each session includes some lecture, some group discussion, a group activity, and individual workbook-based activities.

Groups were cofacilitated by two facilitators who were trained in a 3-day training by the Within My Reach developers. A developer also provided group supervision every other week. Some facilitators were social workers or psychology PhD students, others had no formal training in related fields, but were selected based on their experience with the population and their strong facilitation skills.

In addition to the groups, family support coordinators, often the same people who facilitated some groups, provided four one-on-one case management sessions throughout the program to provide referrals to other needed services (*e.g.*, food assistance, housing) and to reinforce the group curriculum. Meals, on-site childcare, and transportation were provided for free. Women earned up to $200 for attending group and case management sessions. Group assignment was not based on gestational age. Most participants remained with their same cohort throughout the program, however, were able to attend other sessions with a different cohort to make up missed classes. The MotherWise program did not include any clinical care or contraception counseling. It was offered separate from routine perinatal care.

The parent trial reported on long-term (12 and 30 months following baseline) relationship and family stability outcomes and showed that MotherWise improves relationship skills and decreases the number of relationship transitions.^10,11^ A separate subanalysis of a smaller sample reported on birth outcomes and showed that the program was associated with a decreased rate of adverse composite obstetrical outcome.^12^ This subgroup analysis reported here included those participants age 18 or older with a nonanomalous gestation randomized at any time during pregnancy receiving care and delivering at a single safety-net hospital and discharged home with a live infant(s). This study was approved by the Colorado Multiple Institutional Review Board and the University of Denver Institutional Review Board.

Demographic and medical information were abstracted from the electronic medical record (EMR) and stored in Research Electronic Data Capture (REDCap) by trained data abstractors. Race and ethnicity were determined by self-report. The clinically determined estimated due date recorded in the EMR was used to ascertain gestational age. Tobacco and nonprescribed drug use were by self-report and dichotomized as yes or no as any use at any time during pregnancy. Sexually transmitted infections were verified by positive results in the patient's EMR. Hypertensive diseases of pregnancy were defined as gestational hypertension and preeclampsia (chronic hypertension excluded). Medical and mental health comorbidities were defined as any preexisting chronic condition documented by the provider in the EMR. Attendance at postpartum visit was defined as attending a scheduled appointment from 4 to 12 weeks postpartum. Postpartum long-acting reversible contraception (LARC) use was defined as LARC in place at the time of discharge or placed at any postpartum visit upto 12 weeks postpartum.

All analyses were based on intention to treat. Baseline characteristics and all outcomes were compared with appropriate univariate statistics, either Student's *t*-test, chi-squared, or Fisher's exact. Multivariable logistic regression was then used for the primary postpartum outcomes first including all variables with *p* ≤ 0.100 then eliminating those not significant in a backward stepwise fashion. IBM SPSS Statistics 23.0 was used for all data analyses. An *a priori* power analysis was not performed for the primary outcome of this subanalysis as this was a community-based pragmatic trial enrolling all interested women to investigate other long-term relationship and family stability outcomes.

## Results

**FIG. 1.**
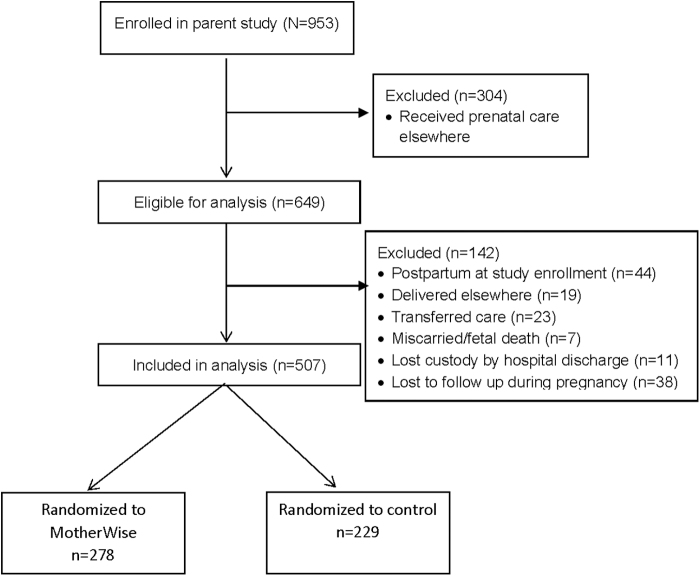
CONSORT diagram.

A total of 953 women were randomized in the parent trial; 507 met inclusion criteria for this study (Fig. 1). Participants were mostly parous (62%), Hispanic (67%), publicly insured (94%) women. There were not any baseline maternal differences in variables measured after randomization (Table 1). More than one-third of each cohort had a medical comorbidity; overall asthma was most common (15.4%), followed by chronic hypertension (7.9%), preexisting diabetes (5.3%), and thyroid disorders (4.9%). More than half of all women had a mental health diagnosis recorded in the medical record, depression being most common (38.5%) followed by anxiety (24.3%), and post-traumatic stress disorder (9.9%). There were not any differences between cohorts in the distribution of the above diagnoses.

**Table 1.** Baseline Maternal Characteristics

	Program, *n* = 278	Control, *n* = 229	*p*
Age, years	28.1 ± 5.8	27.1 ± 6.2	0.073
Parity	1.5 ± 1.6	1.4 ± 1.6	0.391
Medicaid insurance	261 (93.9)	217 (94.8)	0.672
Race/ethnicity			
White, non-Hispanic	37 (13.3)	38 (16.6)	
Black, non-Hispanic	33 (11.9)	30 (13.1)	0.642
Hispanic	195 (70.1)	147 (64.2)	
Other	12 (4.3)	12 (5.2)	
Unknown	1 (0.4)	2 (0.9)	
Spanish-speaking	91 (32.7)	61 (26.6)	0.145
GA at randomization, weeks	23.6 ± 8.3	24.4 ± 8.4	0.274
GA at first prenatal visit	12.3 ± 7.3	12.5 ± 7.6	0.788
Medical comorbidity	112 (40.3)	84 (36.7)	0.406
Mental health comorbidity	152 (55.1)	131 (57.2)	0.631
Tobacco use	49 (17.6)	37 (16.2)	0.661
Drug use	55 (19.8)	46 (20.1)	0.932

All data entered as *n* (%) or mean ± SD.

GA, gestational age.

Of those randomized to MotherWise, 230/278 (82.7%) of women attended at least one class, 79/278 (28.4%) attended all six classes, and overall, on average, participants attended two-thirds of classes (65%). The median gestational age at first workshop was 26.1 weeks (range 4–40 weeks). There were on average seven women in attendance per each MotherWise class.

There were not any differences in antenatal utilization of care between groups (Table 2). Women randomized to MotherWise were more likely to report taking a prescription medicine during pregnancy; selective serotonin reuptake inhibitors were the most common class of medicine in both cohorts (14.2% of all participants) followed by an asthma control agent (13.6%). Participants randomized to MotherWise were more likely to be delivered through cesarean; there were no differences in any other perinatal outcomes (Table 2).

**Table 2.** Antenatal and Birth Outcomes

	Program, *n* = 278	Control, *n* = 229	*p*
No. of routine prenatal visits	11.9 ± 5.4	11.7 ± 5.5	0.599
No. of hospital admissions	3.1 ± 2.3	3.2 ± 2.3	0.689
No. of emergency room visits	0.6 ± 0.9	0.5 ± 1.0	0.483
No. of missed appointments	2.2 ± 2.7	2.3 ± 2.7	0.654
DHS involvement current pregnancy	35 (12.6)	31 (13.5)	0.752
STI during pregnancy	34 (12.2)	40 (17.5)	0.096
Prescription medicine use	164 (59)	106 (46.3)	0.004
Hypertensive disease of pregnancy	67 (24.1)	70 (30.6)	0.103
Gestational diabetes	28 (10.1)	19 (8.3)	0.493
Cesarean delivery	78 (28)	48 (21)	0.050
GA at delivery, weeks	38.8 ± 2.3	38.7 ± 2.0	0.704
Birthweight, grams	3155 ± 573	3118 ± 520	0.450
AGA infant	230 (82.7)	196 (85.6)	0.382
5-minute Apgar	8.7 ± 0.8	8.8 ± 0.6	0.103
NICU admission	47 (16.9)	30 (13.1)	0.235

All data entered as *n* (%) or mean ± SD.

AGA, appropriate for gestational age; DHS, Department of Human Services; NICU, Neonatal Intensive Care Unit; STI, sexually transmitted infection.

On univariate analysis, participants randomized to program were more likely to have LARC in place at the time of discharge from delivery hospitalization (Table 3). They were also more likely to attend their routine postpartum visit (80.2% vs. 72.9%; *p* < 0.053). Among only those women who attended their routine postpartum visit, women randomized to program were more likely to have any contraception in place at that visit, and more likely to receive LARC for contraception (Table 4). There were not any differences in breastfeeding rates at any time (at discharge 83% vs. 82%; *p* < 0.759 and at the postpartum visit 67% vs. 69%; *p* < 0.655).

**Table 3.** Contraceptive Methods at Discharge

	Program, *n* = 278	Control, *n* = 229	*p*
Any contraception at discharge	152 (54.7)	113 (49.3)	0.232
LARC at discharge	66 (23.7)	34 (14.9)	0.012
Sterilization before discharge	32 (11.5)	24 (10.5)	0.713

All data entered as *n* (%) or mean ± SD.

LARC, long-acting reversible contraception.

**Table 4.** Contraceptive Method at 4–12 Weeks Postpartum Among Those Who Attended Postpartum Visit

	Program, *n* = 223	Control, *n* = 167	*p*
Any contraception	177 (79.4)	111 (66.5)	0.004
LARC	108 (48.4)	47 (28.1)	<0.000
Sterilization	22 (9.9)	17 (10.2)	0.918

All data entered as *n* (%) or mean ± SD.

In multivariate regression analyses controlling for antenatal prescription medication use, hypertensive disease of pregnancy, sexually transmitted infection during pregnancy, and mode of delivery, those randomized to MotherWise remained more likely to be discharged from delivery admission with immediate postpartum LARC (odds ratio [OR] 1.84; confidence interval [CI] 1.15–2.96) but were not more likely to attend a postpartum visit (OR 1.49; CI 0.97–2.28). Among those who attended their postpartum visit, the difference in any contraception was no longer significant (OR 1.28; CI 0.76–2.16), however, the difference in LARC use remained (OR 2.19; CI 1.34–3.56).

## Discussion

Our novel prenatal group healthy relationship education program, MotherWise, was associated with double the postpartum LARC use compared with a no-program control group. This effect was seen in the absence of any contraception counseling, without provision of prenatal care or involvement of obstetric providers, only healthy relationship education. Other postpartum behaviors, which might affect a woman's choice of or access to contraception—for example, breastfeeding and attendance at a postpartum visit—were not similarly impacted by the intervention.

MotherWise is not a prenatal care intervention since it lacks the clinical care component that is part of the GPC model. At the same time, MotherWise is based on GPC principles of facilitated group learning, education, and social support. In that vein, our findings could be compared with other studies of GPC and postpartum family planning. In retrospective studies, Hale et al. reported an increased use of any postpartum family planning services in women continuously enrolled in Medicaid choosing Centering Pregnancy, one model of GPC^1^; Trotman et al. reported an increased use of postpartum LARC among adolescents choosing Centering Pregnancy^13^; and Schellinger et al. found increased postpartum LARC use in Hispanic women with gestational diabetes choosing GPC for their prenatal care.^14^ In an observational study, DeCesare et al. also found that women choosing Centering Pregnancy were more likely to return for a postpartum contraception visit and choose LARC.^15^ The results from our randomized trial further strengthens this body of evidence that antenatal group education programs, even those without clinical care, increase the use of effective postpartum LARC.

We theorize it is the element of providing education and fostering empowerment in a supportive group setting during pregnancy that led to a greater use of postpartum LARC for those participants in the MotherWise program. A core concept of Within My Reach and the MotherWise program is “deciding versus sliding.” This concept suggests that we can expect better outcomes when we make clear decisions rather than sliding through transitions or experiences we did not plan for. For many, considering pregnancy as a choice may have been a powerful concept. The longer-term follow-up of the larger sample indicated that those assigned to MotherWise were less likely to have an unintended pregnancy in the 12 months following the intervention.^10^ This finding could be a direct result of greater use of contraception, although future research should replicate these findings and test this association directly.

Our study is a robust randomized controlled trial but must be considered in light of some limitations. We did not differentiate between the implant and intrauterine devices, but rather combined all LARC types as one outcome. Furthermore, we did not follow women for longer than the fourth trimester and do not know rates of continuation or subsequent interval of pregnancy rates. Participants were a cohort receiving care from a safety-net hospital, and results may not be generalizable to other populations. All health outcomes, including the primary outcome of LARC uptake, were determined by report in the EMR and therefore quality and quantity of data were limited to what was recorded and available. As mentioned previously, this study was a planned subanalysis of a larger trial, and therefore may not be adequately powered for the primary outcome; however, our sample size was large and our findings were significant.

Our study has implications for the future research of both GPC and other antenatal psychosocial interventions. We designed a group healthy relationship program without any direct clinical care and yet had a significant impact on the use of postpartum LARCs. Future work should continue to build on and explore how prepregnancy or antenatal education and fostering empowerment can improve women's health.

## Precis

Antenatal group healthy relationship education—without the provision of medical care or contraception counseling—is associated with increased use of postpartum long-acting reversible contraception.

## Abbreviations Used

EMR: electronic medical record
GPC: Group prenatal care
LARC: long-acting reversible contraception
REDCap: Research Electronic Data Capture
